# Target-Based Virtual Screening of Natural Compounds Identifies a Potent Antimalarial With Selective Falcipain-2 Inhibitory Activity

**DOI:** 10.3389/fphar.2022.850176

**Published:** 2022-04-06

**Authors:** Amad Uddin, Sonal Gupta, Taj Mohammad, Diksha Shahi, Afzal Hussain, Mohamed F. Alajmi, Hesham R. El-Seedi, Imtaiyaz Hassan, Shailja Singh, Mohammad Abid

**Affiliations:** ^1^ Medicinal Chemistry Laboratory, Department of Biosciences, Jamia Millia Islamia, New Delhi, India; ^2^ Special Centre for Molecular Medicine, Jawaharlal Nehru University, New Delhi, India; ^3^ Center for Interdisciplinary Research in Basic Sciences, Jamia Millia Islamia, New Delhi, India; ^4^ Department of Pharmacognosy, College of Pharmacy, King Saud University, Riyadh, Saudi Arabia; ^5^ Department of Medicinal Chemistry, Uppsala University, Biomedical Centre, Uppsala, Sweden

**Keywords:** natural compounds, virtual screening, malaria, *in vivo*, *in vitro*, falcipain-2 inhibition

## Abstract

We employed a comprehensive approach of target-based virtual high-throughput screening to find potential hits from the ZINC database of natural compounds against cysteine proteases falcipain-2 and falcipain-3 (FP2 and FP3). Molecular docking studies showed the initial hits showing high binding affinity and specificity toward FP2 were selected. Furthermore, the enzyme inhibition and surface plasmon resonance assays were performed which resulted in a compound ZINC12900664 (ST72) with potent inhibitory effects on purified FP2. ST72 exhibited strong growth inhibition of chloroquine-sensitive (3D7; EC_50_ = 2.8 µM) and chloroquine-resistant (RKL-9; EC_50_ = 6.7 µM) strains of *Plasmodium falciparum*. Stage-specific inhibition assays revealed a delayed and growth defect during parasite growth and development in parasites treated with ST72. Furthermore, ST72 significantly reduced parasite load and increased host survival in a murine model infected with *Plasmodium berghei* ANKA. No Evans blue staining in ST72 treatment indicated that ST72 mediated protection of blood–brain barrier integrity in mice infected with *P. berghei*. ST72 did not show any significant hemolysis or cytotoxicity against human HepG2 cells suggesting a good safety profile. Importantly, ST72 with CQ resulted in improved growth inhibitory activity than individual drugs in both *in vitro* and *in vivo* studies.

## 1 Introduction

Malaria is one of the endemic tropical parasitic diseases, caused by blood-borne Apicoplexan parasite *P. falciparum* especially in the developing world (Aneja et al., 2016; Kumar et al., 2021). Irrespective of the efforts made to control malaria under its various eradication programs, in 2019, the World Health Organization (WHO) estimated 229 million cases of malaria worldwide and 409,000 deaths. In 2019, children aged under 5 years are the most vulnerable group as they accounted for 67% (274,000) of all malaria deaths worldwide (World malaria report, 2019; Valério Lopes et al., 2021). The emergence and spread of the resistance to the commonly available and inexpensive drugs limited the efficiency of antimalarial therapies resulting in an increase of the mortality rate (Gelb, 2007; Uddin et al., 2021). To combat the spread of multidrug resistance in *Plasmodium* strains, the WHO recommended artemisinin-based combination therapies (ACTs). However, there have been reports on decreased susceptibility of the parasite to ACTs in the Greater Mekong Subregion (GMS), followed by ACTs’ failure leading to a worrisome situation (Nsanzabana, 2019). Therefore, it becomes imperative to explore newer strategies for the control and eradication of malaria.

Among various drug targets, the proteases of *P. falciparum* act as the attractive target for most of the drugs playing a crucial role in growth and development of the parasite during its life cycle (Pandey, 2003). A complete genome of *P. falciparum* consists of 33 open-reading frames that encode cysteine proteases and a family of four cathepsin L–like papain proteases collectively known as falcipains (Kerr et al., 2009a). Falcipain-2 (FP2) and falcipain-3 (FP3) are key papain-family (C1) clan CA trophozoite cysteine proteases which lie in the digestive food vacuole that cleaves host hemoglobin (native and denature) and is also responsible for erythrocyte rupture (Shenai et al., 2000; Sijwali et al., 2001b, 3; Hogg et al., 2006). The inflation of malarial parasite inside the infected red blood cell is proportionate to the rate of its food assimilation (Goldberg et al., 1990). Cysteine proteases of *Plasmodium* are implicated with several biological processes such as rupture of membranes, degradation of Hb, protein trafficking, and host cell invasion (Lehmann et al., 2014; Melo et al., 2018). Thus, the growth of malarial parasites is measured by the interference of key indicator protein FP2 (Liu et al., 2006). Among falcipains, the most expressed and well-studied one is FP2 which is an attractive target for the discovery of antimalarial chemotherapy (Fagnidi et al., 2018).

The traditional drug discovery approach of high-throughput screening (HTS) of a large chemical library for the identification of new antimalarial drug leads is time-consuming and resource-intensive (Keshavarzi Arshadi et al., 2019). To minimize the cost and time for a drug development process, computer-aided drug design (CADD) is one of the extensively used methods (Li et al., 2009; Lima et al., 2018). This method employs *in silico* techniques which allow us to analyze a large chemical library in a short period of time to identify the hits based on their interaction with the target protein (Mao et al., 2021). It also allows analyzing the stability of the protein–ligand complex using simulation tools and prediction of the drug-like properties. Virtual screening is one of the promising computer-aided techniques in identifying new leads for drug development (Shoichet, 2004; Ravindranath et al., 2015; Fradera and Babaoglu, 2017; Hernández-González et al., 2021; Rajguru et al., 2022). Structure-based virtual screening (SBVS) or target-based virtual screening (TBVS) is commonly used for the identification of potential compounds preferentially binding to a drug target (Rosenthal, 2003; Hernández González et al., 2017; Gau et al., 2018; Maia et al., 2020; Rana et al., 2020; Dash et al., 2021). SBVS involves computational methods, including molecular docking and dynamic simulations of receptor–ligand and the complexes (Desai et al., 2004; Rajguru et al., 2021). The availability of several crystal structures of FP2 in complexes with inhibitors (PDB ID: 3BPF, 2OUL, 6JW9, etc.) opened a newer avenue to discover its inhibitors as potent antimalarials through virtual screening based on their structures from natural/synthetic libraries (Wang et al., 2014; Rajguru et al., 2021, 2022). In the present study, a natural library of _˜_90,000 compounds from the ZINC database was screened through SwissADME for their likelihood of drug molecules based on physicochemical and ADMET properties. Furthermore, we used InstaDock to identify high-affinity binding partners of FP2 by utilizing the molecular docking approach. The compounds showing high binding affinity and specificity toward FP2 were selected. We identified three hit compounds, viz., ZINC5434062 (NT23), ZINC12900664 (ST72), and ZINC5434059 (NT18), as potential FP2 inhibitors and subjected them to various biochemical studies with the purified FP2. The compound ST72 with EC_50_ values of 2.8 and 6.7 µM against CQ^S^ and CQ^R^ strains, respectively, reduced significant parasite load and increased host survival in a murine model infected with *P. berghei* ANKA. Thus, the study revealed ST72, a natural FP2 inhibitor with potent antimalarial activity, for further biological investigations.

## 2 Materials and Methods

### 2.1 Computational Resources

A well-defined computational pipeline of drug design and discovery approach uses different bioinformatics software, including InstaDock and Discovery Studio (Mohammad et al., 2021). PyMOL was used for visualization. Web resources such as RCSB-Protein Data Bank (PDB), the ZINC database (https://zinc12.docking.org/pdbqt/), SwissADME (http://www.swissadme.ch/, Daina et al., 2017), CarcinoPred-EL (Ai et al., 2018), VMD (Humphrey et al., 1996), and QtGrace are utilized in data evaluation, retrieval, and analysis purposes (Zhang et al., 2017). The structural coordinates of FP2 and FP3 were downloaded from PDB (PDB ID: 3BPF and 3BPM, respectively). All cocrystallized hetero atoms, including water molecules and cocrystallized ligand E64, were pulled out from the original coordinates. Finally, the structure of protein was prepared for virtual screening in the SPDBV tool by remodeling missing residues, adding H-atoms to polar atoms, and giving appropriate atom types. The library of natural compounds was retrieved from the ZINC database and subsequently processed in InstaDock.

#### 2.1.1 Physicochemical Properties of Compounds

The compound library downloaded from the ZINC database (https://zinc12.docking.org/pdbqt/ *.*) was checked for the physicochemical properties while using the pkCSM and SwissADME web servers (Irwin and Shoichet, 2005). We have checked each compound for whether they are following Lipinski’s rule of five or not (Lipinski, 2000). We have applied the Pan-assay interference compounds (PAINS) filter to remove compounds having specific patterns that show a higher tendency of binding toward multiple biological targets. We have also predicted the ADMET (absorption, distribution, metabolism, excretion, and toxicity) properties to select only those compounds showing good ADMET and drug-like properties. Only compounds showing a set of good physicochemical properties were selected for further exploration.

#### 2.1.2 Molecular Docking Studies

Virtual screening of filtered compounds by molecular docking was employed to quickly determine the affinity of each compound toward FP2 and FP3. This screening was performed while utilizing the molecular docking approach to find out compounds based on their affinity and interaction with FP2 and FP3 (Uddin et al., 2020). The docking was carried out using InstaDock with a blind search space of a grid size of 73, 59, and 59 Å, centralized at -47.49, -12.34, and -12.62 for *X*-, *Y*-, and *Z*-axes, respectively. The spacing of the grid size was 1.00 Å with default docking parameters. The docking output was filtered based on the energy values, and then all docking poses of each compound were generated for the interaction analysis while utilizing PyMOL and Discovery Studio Visualizer.

#### 2.1.3 Interaction Analysis

The docking output of the filtered compounds was generated for their possible conformations and explored while utilizing PyMOL and Discovery Studio Visualizer. The close interactions between the compounds and FP2 were mapped by depicting the polar contacts within 3.5 Å of distance in PyMOL. To know the interactions formed between the compounds and FP2, the software Discovery Studio Visualizer was utilized. We have selected only those compounds found to interact with the critical residues within the binding pocket of FP2 through the interaction analysis. We have chosen only those compounds showing specific and close interactions with FP2, including Cys42, and mimicking the binding pose of the reference compound E64, a cocrystallized known FP2 inhibitor.

### 2.2 Biochemical Inhibition of FP2 and Antimalarial Activity of Lead Compounds

#### 2.2.1 Enzyme Inhibition Assays

The constructs of FP2 and FP3 were kindly gifted by Dr. K. C. Pandey, National Institute of Malaria Research, ICMR, India. The FP2 and FP3 recombinant proteins were purified and refolded as described previously (Sijwali et al., 2001b). Inhibition of FP2 and FP3 by NT23, ST72, and NT18 was observed by a spectrofluorometer. Briefly, recombinant FP2 and FP3 were incubated for 30 min with NT23, ST72, and NT18 at concentrations 5, 12.5, and 25 µM in 100 mM sodium acetate (pH 5.5) with 8 mM dithiothreitol (DTT). FP2 and FP3 substrate Z-Phe-Arg-AMC was added just before the measurement (Vigyasa Singh et al., 2020). Samples were measured for 10 min at RT at Ex = 355 nm and Em = 460 nm to calculate the percentage of hydrolysis of peptide substrate Z-Phe-Arg-AMC which reflects the percent inhibition of falcipains. The experiment was done in triplicate.

#### 2.2.2 Surface Plasmon Resonance

SPR technique was used to determine the NT23, ST72, and NT18 interactions with FP2 and FP3. SPR was done by using Auto Lab Esprit SPR. Briefly, the surface of charged SPR chip was immobilized with 10 µM concentration of FP2 and FP3. Different concentrations (6.25–400 µM) of compounds were injected over the chip surface for the interaction analysis (Jain et al., 2020b). Association and dissociation time of 300 and 150 s was fixed. PBS (1X) pH 7.4 buffer was used for immobilization and binding. NaOH solution (50 mM) was used for regeneration of surface of the sensor chip. Data were analyzed by using Auto Lab SPR Kinetic Evaluation software.

#### 2.2.3 Hemozoin Inhibition Assay

Parasite culture synchronized at ring stage (6% parasitemia) was treated with various concentrations of ST72 (0.31, 0.62, 1.25, 2.5, 5, 10, 20, and 40 µM). Parasite suspension was seeded into a 96-well flat bottom plate in triplicate. After 30 hrs, 2.5% SDS made in 0.1 M sodium bicarbonate pH 8.8 was added to the samples and incubated at room temperature for 30 min. The pellet obtained after centrifugation was resuspended in 5% SDS and 50 mM NaOH incubated for 30 min. The amount of monomeric heme was quantitated at 405/750 nm as described previously (Men et al., 2012).

#### 2.2.4 Parasite Culture

Chloroquine-sensitive (CQ^S^) (3D7) and chloroquine-resistant (CQ^R^) (RKL-9) *P. falciparum* strains were cultured in fresh O+ erythrocytes in complete RPMI 1640 (supplemented with 10 mg/L gentamicin, 2 g/L sodium bicarbonate, 50 mg/L hypoxanthine, and 0.5% Albumax I) according to Malaria Research and Reference Reagent Resource Center (MR4) guidelines (Mphande et al., 2008). Thin blood smears stained with Giemsa were prepared for routine monitoring. Synchronization of parasite stage was done with the help of sorbitol for the selection of rings and Percoll for the selection of trophozoites.

#### 2.2.5 SYBR Green I–Based Fluorescence Assay

DNA-specific dye SYBR Green I (Invitrogen, Carlsbad, United States) was used to perform the growth inhibition assay to calculate the inhibitory effect of compounds against the parasite *P. falciparum* 3D7 (Makler et al., 1993). The selected compounds from the *in silico* study, NT23, ST72, and NT18, were selected for further growth inhibition assays. Initially, parasites (3D7 and RKL-9 strains of *P. falciparum*) at ring stage with 1% parasitemia and 2% hematocrit were treated with different concentrations (0.3–20 µM) of NT23, ST72, and NT18; untreated parasites were used as negative control. Samples were seeded in 96-well microtiter plates and incubated at 37°C for 60 h s post-treatment. After incubation, the plates were sealed with Parafilm and kept at -80 °C. 96-well plates were thawed at 37 °C prior to analysis. Lysis buffer (5 mM EDTA, 0.008% saponin, 0.08% Triton X-100, and 20 mM Tris, pH 7.5) was freshly prepared, and 0.2 μL/ml SYBR Green I per mL was added to it. 100uL of lysis buffer was put in each well and kept at 37°C for 3 hrs in dark. The fluorescence intensity was observed at Ex = 485 nm and Em = 530 nm spectrum. Percentage growth inhibition was estimated as follows: % inhibition = [1—% parasitemia (treatment)/% parasitemia (untreated control)] *100. Data were expressed as mean ± SD. The effective concentration (EC_50_) values of NT23, ST72, and NT18 were determined (GraphPad Prism 8 software).

#### 2.2.6 Stage-Specific Assay

The standard asexual blood stage assay was performed by exposing synchronous parasite culture to different concentrations of ST72. Briefly, the parasite culture was synchronized at schizont stage by 65% Percoll treatment followed by synchronization at ring stage with 5% sorbitol to obtain pure ring stage culture. These parasites were plated in a 96-well plate at 0.8% parasitemia and 2% hematocrit and treated with ST72 at rings (12–14hpi), trophozoites (32–34hpi), or schizonts (38–40hpi). Stage-specific treatment was done for 6 h s, and compounds were removed through washing with complete media after each treatment. For the stage-specific effect, the growth inhibition was monitored 60 hrs post-treatment where parasite developed into the trophozoite stage. Parasite survival for 72 hrs after treatment was assessed by the SYBR Green method. The effective concentration (EC_50_) values were obtained for each treatment from growth inhibition data using Prism 8.0.1, GraphPad. For the combination assay, ST72 or that in combination with CQ at various concentrations of ST72 (2–16 µM) + CQ (10–80 nM) was setup according to the fixed ratio method (Murithi et al., 2020). The plates were incubated for 72 hrs at 37°C. The parasitemia was determined by the SYBR Green method as described above (Gupta et al., 2019). The combination index (CI).

$$\left(EC_{x}\;\mathrm{of}\;\mathrm{Drug A with Drug B}/EC_{x}\;\mathrm{of}\;\mathrm{Drug A}\right)+\left(EC_{x}\;\mathrm{of}\;\mathrm{Drug B with Drug A}/EC_{x}\;\mathrm{of}\;\mathrm{Drug B}\right).$$

#### 2.2.7 Hemolytic Activity

The hemolytic assay was done to observe the effect of compound ST72 on human RBCs (Asghar Ali et al., 2021). Briefly, erythrocytes at 10% (v/v) were washed with 1× PBS (pH 7.4), and the cell suspension was treated with ST72 at different concentrations (1.8–1,000 µM) at 37°C for 2 hrs. The samples were centrifuged, and hemoglobin absorbance was taken by a Varioskan microplate reader (Thermo Fischer Scientific) at 415 nm to obtain the percentage of RBC lysis. 0.15% saponin was used as a positive control for the experiments. The percentage of RBC lysis was calculated as follows: % RBC lysis = (OD_415nm_ sample− OD_415nm_ PBS)/(OD_415nm_ Triton X-100 1%− OD_415nm_ PBS).

#### 2.2.8 Cytotoxicity Assay

The colorimetric MTT assay was performed to check the cytotoxicity of ST72 toward human liver cancer cell line (HepG2) cells (Vigyasa Singh et al., 2020). HepG2 cells were cultured in DMEM enriched with 10% fetal bovine serum with 10% penicillin–streptomycin solution at 37°C in a humidified atmosphere of 5% CO_2_. A total of 1×10^4^ HepG2 cells were seeded in triplicate in 96-well plates, and the cells were treated with ST72 at concentrations ranging from 2.5 to 1000 μM for 48 h s at 37°C. Freshly prepared MTT at 5 mg/ml was added to the samples and incubated at 37°C for 4–5 hrs. DMSO was added to solubilize the formazan crystals. A microplate reader (Varioskan Thermo Fischer Scientific) was used for taking the absorbance at 570 nm, and all the experiments were done in triplicate.

#### 2.2.9 *In Vivo* Antimalarial Activity

The animal studies were conducted at Jawaharlal Nehru University. All experimental procedures conducted on the animals were in accordance with the rules and regulations set by Institutional Animal Ethics Committee (IAEC) code no. 35/2019. Female Balb/c mice, of 6–8 weeks of age weighing ∼30 g, were used in this study to determine the antimalarial activity of ST72. Briefly, the animals were randomly distributed into four groups (n = 4): control (untreated), ST72, chloroquine (CQ), and ST72 + CQ, and infected with 1×10^6^ *Plasmodium berghei* ANKA parasites in 1X PBS intraperitoneally. Thin smears were made from the tail blood of mice to check the infection. Post-infection, the compounds were administered in *P. berghei*–infected mice intraperitoneally daily for eight consecutive days. To determine parasitemia, blood from the tail of mice was taken each day and thin smears were prepared. The parasitemia was determined by counting 2000 erythrocytes in Giemsa-stained smears using a light microscope at ×1000 magnification. The mean survival time for mice in each group was estimated by calculating the average survival time, in days post-inoculation over a period of 24 days. To measure vascular permeability in the brain of mice due to increased parasite load, the Evans blue permeability test was performed as described previously (Leten et al., 2014). Briefly, 100 µL of 2% Evans blue was injected into the lateral tail vein in mice on day 7 after infection with *P. berghei* ANKA and euthanized 1 h s later. Brains were removed for assessment of Evans blue dye impregnation.

#### 2.2.10 Statistical Analysis

To analyze the mean values obtained for the treatment and control, one-way analysis of variance (ANOVA) was used. Dunnett’s test was used to compare the treatment and control, and statistical significance was set at **p < 0.01 *vs*. control. *p* > 0.05 implies non-significance.

## 3 Results

### 3.1 *In Silico* Studies

#### 3.1.1 Molecular Docking–Based Virtual Screening

**FIGURE 1:**
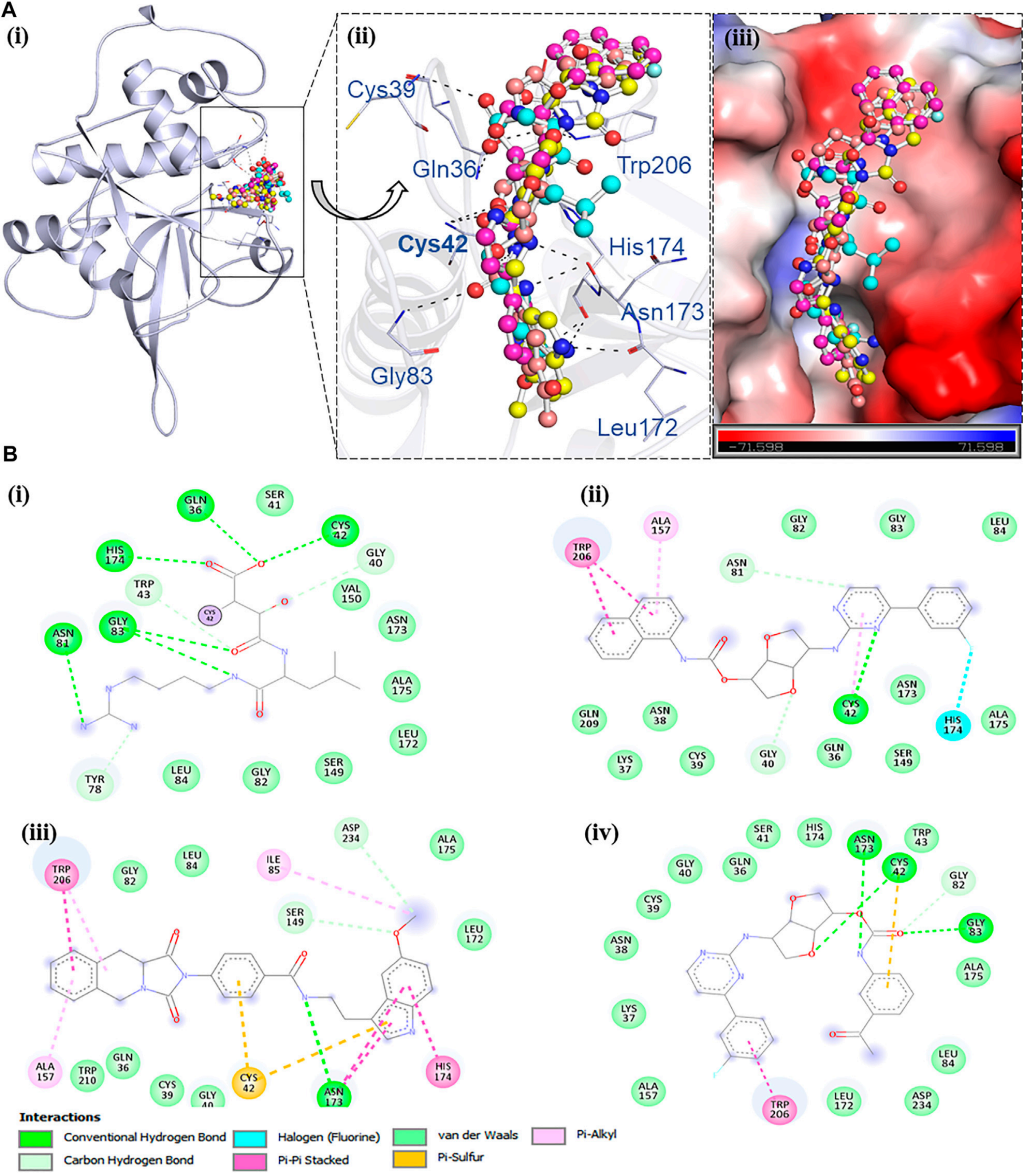
Structural representation of FP2 complexed with NT23, ST72, NT18, and E64. **(**A (i)**)** Representation of the cartoon *structure of* FP2 with the lead compounds. (A (ii)) Zoomed view indicating the interaction of FP2 with E64 (cyan), NT23 (magenta), ST72 (yellow), and NT18 (red). (A (iii)) Surface potential view of the FP2 pocket occupied by the selected compounds and the electrostatic potential was generated using PyMOL. **(B)** Two-dimensional structural representation of binding pocket residues of FP2 and their interactions with (B (i)) E64, (B (ii)) NT23, (B (iii)) ST72, and (B (iv)) NT18.

The compounds having appreciable binding scores with FP2 were selected and studied further in the molecular docking–based virtual screening attempt. Based on the docking analysis, initially, we have selected the top 30 compounds showing appreciable binding affinities (−9.1 to −11.5 kcal/mol) toward FP2. These 30 compounds were further docked with FP3 to identify those compounds showing *appreciable binding* affinity toward FP2 (Supplementary Tables S1, S2). We selected the top 10 compounds showing highest binding affinities with FP2 as compared to FP3. Furthermore, a detailed analysis of these 10 compounds was performed for their specific interactions toward the FP2 binding site while utilizing PyMOL and Discovery Studio Visualizer (Supplementary Table S3; Figure 1A).

#### 3.1.2 Physicochemical Properties of Compounds

The physicochemical properties of all the compounds from the ZINC library were calculated by using SwissADME and Discovery Studio. About 32,902 compounds from the parental library passed Lipinski’s rule of five and had zero PAINS pattern with a good bioavailability score. Here, we have shown those compounds which were finally selected after docking (NT23, ST72, and NT18) along with the known FP2 inhibitor E64, which show a set of good physicochemical properties (Shoichet, 2004). The calculated properties of NT23, ST72, and NT18, along with a known FP2 inhibitor E64, are shown in Supplementary Table S3. We can see that NT23, ST72, and NT18 show a similar set of drug-like properties to E64.

#### 3.1.3 Interaction Analysis of Lead Compounds

For interaction analysis, all possible docking conformations were split from the out files of the selected ten compounds. Among all docked conformers’ analysis, three compounds, NT23, ST72, and NT18, were found to interact with active site amino acid residues (Q36, C42, H174, N204, and W206) (Kumar et al., 2004), within the binding pocket of FP2 where the cocrystallized E64 bound (PDB ID: 3BPF). The detailed binding pattern and interacting residue of the finally selected three compounds NT23, ST72, and NT18 along with E64 are shown in Figure 1A and Supplementary Figures S1, S2.

All compounds binding in the FP2 binding pocket were checked for their detailed interactions with the critical residues of the protein. The interaction analyses was performed to understand the exact type of non-covalent interaction and the various types. Detailed interactions and their types between the compounds and the protein are shown in Figure 1B. It is evident that all three compounds NT23, ST72, and NT18 interact within the binding site of FP2 and share the common interactions of the previously known FP2 inhibitor.

### 3.2 *In Vitro* Biochemical Inhibition and Antimalarial Activity of Selected Compounds

#### 3.2.1 Enzyme Inhibition Study

**FIGURE 2:**
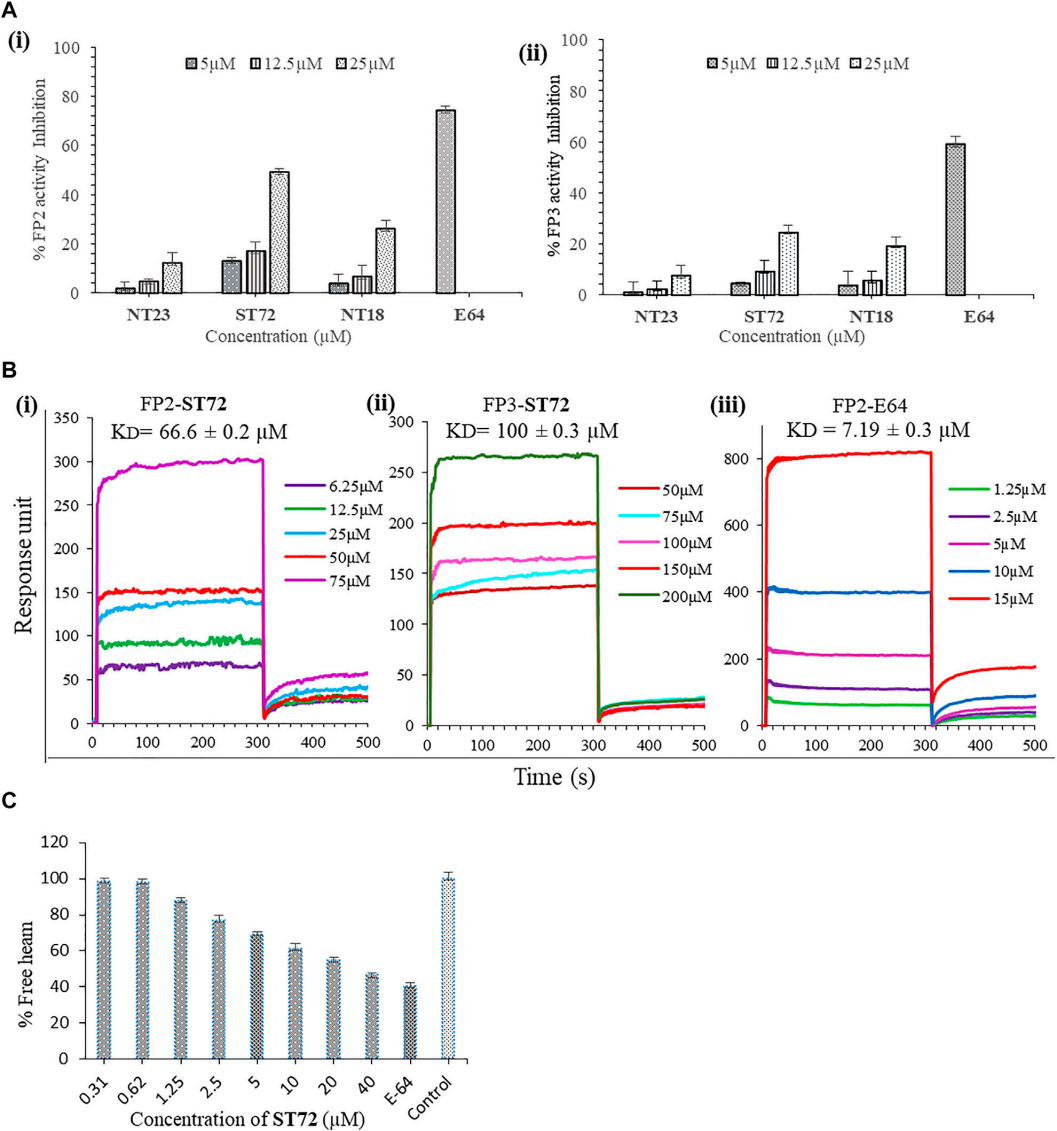
**(A)** The bar graph represents enzyme inhibition activity of NT23, ST72, and NT18 for (i) FP2 and (ii) FP3. Hydrolysis of substrate Z-Phe-Arg-AMC was calculated. The graph shows increased percentage inhibition of FP2, indicating attenuation of hydrolysis of substrate peptides was calculated by fluorescence intensity. Data were expressed as mean values ± SD. The experiments were carried out in duplicate. **(B)** Concentration-dependent real-time sensograms for SPR-based biomolecular interaction between FP2 (i) and FP3 (ii) with lead compound ST72. Recombinant proteins were immobilized on the surface of charged SPR chip. The experiment was performed with increasing concentrations of ST72 at association and dissociation time of 300 and 150s. E-64 (iii) was used as a positive control showing strong interaction with FP2. **(C)** Quantification of hemozoin inhibition by calculating percent free heme in ST72-treated parasite culture. Synchronized ring-stage parasites exposed to ST72 at different concentrations (0.31–40 µM) and percent free heme were estimated.

Recombinant FP2 and FP3 were prepared as described earlier (Sijwali et al., 2001b, 3, Sijwali et al., 2001a, 2). Compounds NT23, ST72, and NT18 were purchased from MolPort, Latvia. The interaction of FP2 with identified compounds NT23, ST72, and NT18 from in silico studies was further validated by enzyme inhibition assays. Enzymatic activity of recombinant FP2 and FP3 with cysteine proteases-known substrate Z-Phe-Arg-AMC was measured by spectrofluorometry. The enzyme falcipain is responsible for cleavage of peptide substrate and release of fluorogenic AMC. The results showed the percentage inhibition of FP2 and FP3 in a dose-dependent manner by NT23, ST72, and NT18 at different concentrations (5, 12.5, and 25 μM). At highest concentration used for the experiments, i.e., 25 μM for NT23, ST72, and NT18, the percent enzyme inhibition was found to be 12, 49, and 26%, respectively, against recombinant FP2. However, a known falcipain inhibitor E64 (5 µM) used as a positive control for the experiments showed >70% inhibition against FP2. No significant inhibition of FP3 was observed upon treatment with above compounds even at a higher concentration of 25 µM (Figure 2A). Together, our results depicted that ST72 has a significant impact on FP2 activity as compared to other tested compounds. Therefore, expansion in the percentage inhibition of FP2 with ST72 treated samples showed their function to block FP2.

#### 3.2.2 Interaction Analysis of Lead Compounds With Recombinant Falcipain Proteins

Here, we presented surface plasmon resonance (SPR) as a first step in the search of new compounds that interact with falcipain 2 specifically. SPR allows for fast analysis of interacting ability between the macromolecule and the analyte, thereby permitting the determination of association and dissociation constants important for the selection of the inhibitor or binder (Holanda et al., 2020). Here, we determined the binding affinity of FP2 and FP3 with NT23, ST72, and NT18 and studied the protein–drug interaction using surface plasmon resonance (SPR). Different concentrations of NT23, ST72, and NT18 were injected over immobilized recombinant FP2 and FP3. E-64 was used as a positive control. We found that ST72 at increasing concentrations ranging from 6.25 to 75 µM directly interacts with recombinant FP2. The concentration-dependent real-time sensograms for the interaction analysis of ST72 with the recombinantly purified FP2 and FP3 demonstrated affinity constant values (KD) to be 66.6 ± 0.2 μM and 100 ± 0.3 μM, respectively, indicating strong binding to FP2 (Figure 2B). SPR analysis of other compounds with FP2 and FP3 showed weak interaction even at a higher concentration of 400 µM (Supplementary Figure S3). Although FP2 and FP3 shared considerable sequence homology, their binding with the lead compound showed the significant difference as depicted by SPR. Thus, it could be due to low affinity between recombinant protein and compound which depends on various factors such as complementarity of binding surfaces, correct confirmation of proteins, and stability of compounds in the buffer. Our SPR analysis measures the strength of interaction of selected compounds with both FP2 and FP3, indicating that ST72 has high binding affinity for FP2 protein than FP3.

#### 3.2.3 Hemozoin Inhibition Study

Hemoglobin degradation produces ferric heme which is a byproduct and proves to be lethal to both the malarial parasites and the host cells (Huy et al., 2002, 2013; Rowe et al., 2009; Gorka et al., 2013). Consequently, malarial parasite for its protection resorts to crystallization into hemozoin, converting toxic heme into non-toxic metabolites. Hemozoin is a malaria pigment, which is insoluble in water (Francis et al., 1997; Huy et al., 2002). Spectrophotometric analysis was performed to examine free monomeric heme acquired from hemozoin. For the quantification of monomeric heme, synchronized parasites at ring stage were incubated with ST72 and followed up to the schizont stage. Parasites treated with ST72 showed dose-dependent decreased percentage of free monomeric heme as compared to untreated control. At 40 µM concentration, ST72 showed 46.3%, whereas E-64 showed 40.4% free heme at 5 µM concentration (Figure 2C). The free monomeric heme was quantified at 405/750 nm absorbance which later correlated with the reduction in hemoglobin hydrolysis. Since ST72 is predicted to inhibit activity of falcipain that would result in less production of free heme, crystallization of hemozoin is vital for persistence of malarial parasite that creates an interest toward the development of novel antimalarial drugs (Gorka et al., 2013; Vigyasa Singh et al., 2020).

#### 3.2.4 Antimalarial Activity by SYBR Green I–Based Fluorescence Assay

**FIGURE 3:**
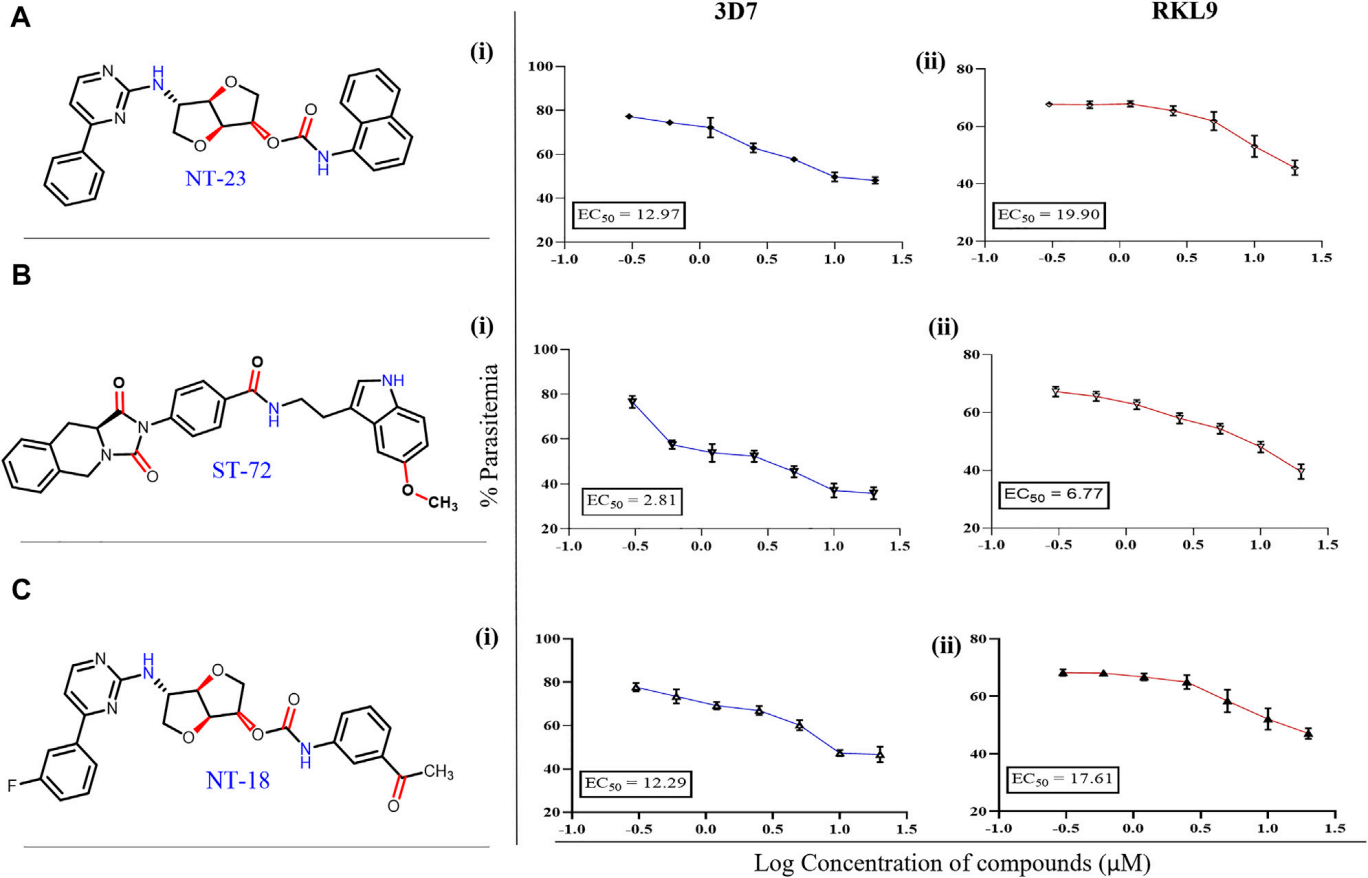
**(A–C)** Growth inhibitory effect of compounds NT23, ST72, and NT18 on 3D7 and RKL-9. The parasite culture synchronized at ring stage was treated with different concentrations (0.3–20 µM) of these compounds for 72 h s, and the percent growth inhibition was estimated by the SYBR Green fluorescence assay. IC_50_ values of each compound in 3D7 (i) and RKL-9 (ii) were evaluated by plotting growth inhibition values against the log concentration of these compounds. The experiment was done in triplicate, and the results were shown as mean values ± SD.

Antimalarial potential of naturally inspired compounds being identified from the available natural compounds’ database is previously reported (Jahan et al., 2015). The therapeutic efficiency of these compounds as antimalarial is still questioned because of their limited mechanistic information, the stability of the compound, and the low potency of antimalarial. Therefore, there is a pressing need to identify naturally inspired compounds against key parasite proteins like falcipain, for effective antimalarial. In the present study, the antiplasmodial potency of lead compounds NT23, ST72, and NT18 from the ZINC database was assessed on *P. falciparum* chloroquine-sensitive and -resistant strains by the standard growth inhibition assay. One complete intraerythrocytic growth cycle of *P. falciparum* was examined after the treatment. The DNA-specific dye SYBR Green I assay was performed to determine parasite growth during intraerythrocytic stages (Uddin et al., 2020). As observed, the effective concentration (EC_50_) of compounds showed variable antimalarial activity against both chloroquine-sensitive (3D7) and chloroquine-resistant (RKL-9) strains. EC_50_ values of ST72 depicted twofold higher inhibitory activity against chloroquine-sensitive 3D7 (EC_50_ = 2.8 µM) as compared to the chloroquine-resistant parasite (EC_50_ = 6.7 µM) (Figure 3B (i-ii)). The compounds NT23 and NT18 showed antimalarial potency against 3D7 with EC_50_ values of 12.97 and 12.29 µM, respectively. In RKL-9, EC_50_ values of NT23 and NT18 were found to be higher as compared to that of 3D7 (Figures 3A,C). Together, our results indicate that, of all the compounds tested, ST72 has the lowest EC_50_ value against sensitive and resistant strains. Therefore, based on the preliminary study, we stated that ST72 could be a potential antimalarial lead compound against both sensitive and resistant parasites.

#### 3.3.5 Maximal Inhibitory Effect of ST72 on Parasite Growth at Trophozoite Stage

**FIGURE 4:**
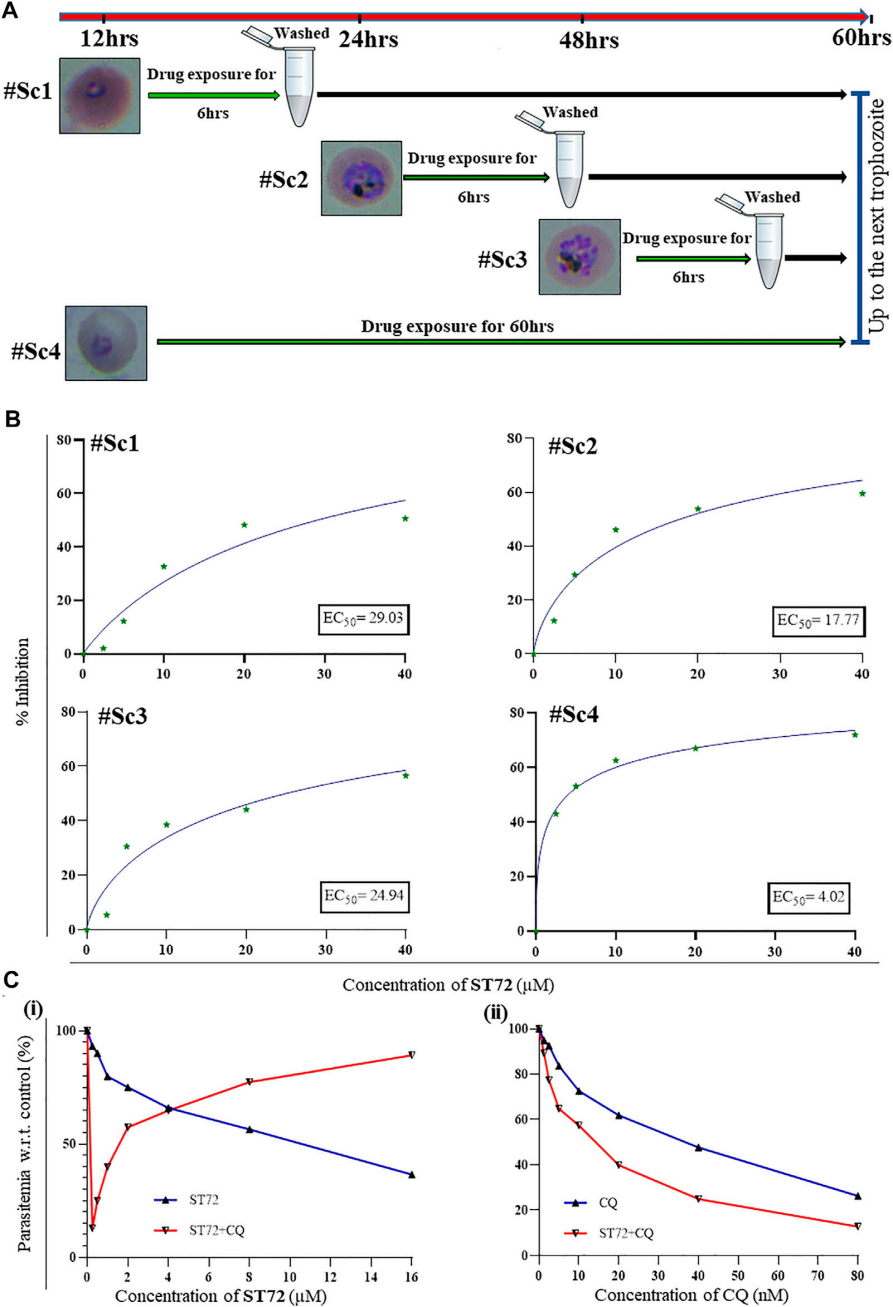
Stage-specific susceptibility profile for ST72 against 3D7. **(A)** Schematic representation showing parasites at different stages were exposed to ST72 for 6 hrs. Growth was monitored at 60 h s post-treatment assessed by SYBR Green. **(B)** The graphs indicate the percentage growth inhibition by ST72 in a dose-dependent manner. IC_50_^6hrs^ values were calculated using GraphPad software. **(C)** Drug combination assay exhibiting the effect of ST72 on growth of malarial parasites when tested in combination with chloroquine (CQ) or both drugs alone. Graphs (i) and (ii) represent growth patterns of parasites treated with these compounds individually and in combination, for 72 hrs. The concentrations of individual compounds used in the combination assays are included for each data point.

During the asexual phase of life cycle, the parasite must undergo progression through different stages, i.e., rings, trophozoites, and schizonts. The quantitative assessment of susceptibility of different stages to ST72 treatment by an *in vitro* intraerythrocytic stage-specific assay was done (Matthews et al., 2020). By light microscopy, different periods of exposure at distinct developmental stages were determined, indicating all these asexual stages were described (Figure 4A). In these experiments, the tightly synchronized parasites at each stage were exposed to compound ST72 for a 6 h s period followed by washing after each exposure period which led parasites to grow in the absence of compound till the end of the assay. The parasite growth was monitored at the same time point, i.e., 60 h s. EC_50_^6hrs^ values determined for each treatment depicted cytotoxicity of the compound ST72 in a stage-specific manner. As observed in our results, ST72 showed pronounced variations in EC_50_^6hrs^ values through rings to schizonts with maximal effect in the metabolically active stage, i.e., trophozoites. In contrast, synchronized rings treated with different concentrations of the compound for 72 h s showed cytostatic effect with EC_50_^72hrs^ of 4 µM. The increased activity of lead compound at trophozoite stage can be linked with hemoglobin degradation within digestive vacuoles as observed with several antimalarials (Murithi et al., 2020). Cysteine proteases such as FP2 and FP3 are important for hemoglobin catabolism in malarial parasites, thereby acting as attractive targets for antimalarial therapy. Biochemical studies with recombinant proteins have already revealed ST72 specifically binds to FP2 and inhibits its activity. Together, these results showed the FP2-targeting effect of ST72 might lead to inhibition of parasite growth during intraerythrocytic stages (Figure 4B).

#### 3.3.6 ST72 Combination Study With CQ

To combat the problem of antimalarial resistance, there is a pressing need for development of new drug combinations that can target multiple pathways in malarial parasites. Here, we used the systematic approach to validate possible drug combinations of ST72 with known antimalarial chloroquine (CQ) (Gupta et al., 2019). The effect of compounds alone or in combinations was assessed on the growth of parasite. The total parasitemia with respect to untreated control was estimated 60 h s post-treatment. We evaluated the effect of compounds on parasite growth by the fluorescence-based SYBR Green I method. The results depict EC_50_ values were calculated for each of the drugs alone and in combination relative to individual drugs. The results for combination assays showed synergistic interactions between chloroquine and ST72 with combination index (CI = 0.5), indicating that these two compounds together exhibit full potential with the maximal inhibitory effect on parasite growth (Figure 4C).

#### 3.2.7 *In Vivo* Antimalarial Activity and Combination Study of ST72

**FIGURE 5:**
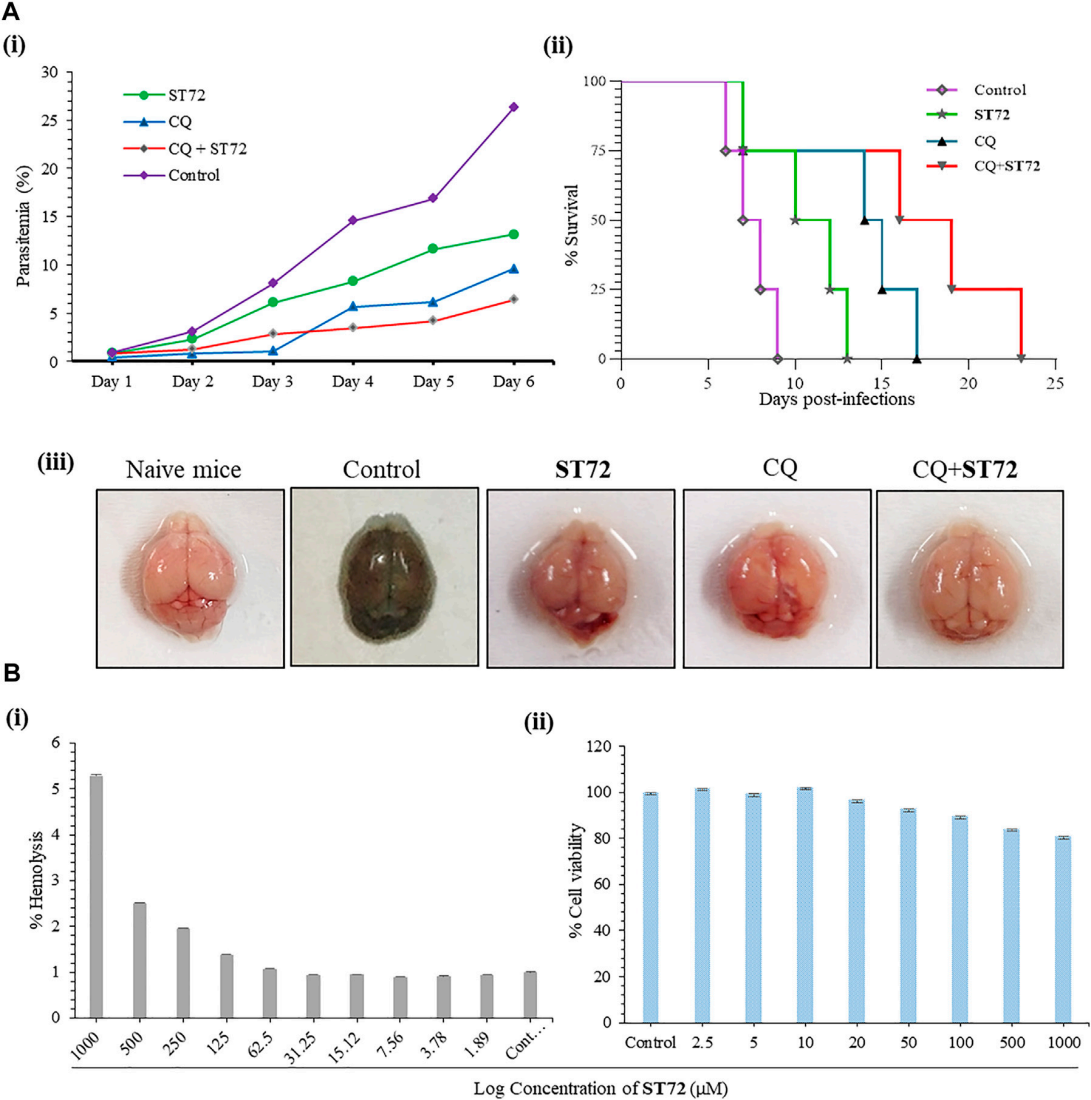
**(A)** *In vivo* antimalarial activity of ST72 was evaluated against *P. berghei* ANKA in Balb/c mice. ST72 (2.5 mg/kg body weight; n = 4), chloroquine (6 mg/kg body weight; n = 4), and combination (2.5+6 mg/kg body weight; n = 4) were administered intraperitoneally in *P. berghei* ANKA–infected Balb/c mice for six consecutive days. The untreated group of mice was taken as control. Thin blood smears were made up to day 6 post-infection. (A (i)) Graph showing percent reduction in parasitemia in ST72-treated mice or in combination with CQ. (A (ii)) The survival of mice was observed up to 24 days post-infection. The graph showed better survival in ST72-treated mice as compared to control percentage survival of mice. (A (iii)) Evans blue staining of brains of *P. berghei*–infected mice 7 days post-infection. **(B** (i)**)** A graph showing the effect of ST72 in a dose-dependent manner on human RBCs as analyzed by the hemolytic assay. No significant effect was observed in the treated samples up to 500 µM concentration of ST72. (B (ii)) The colorimetric MTT assay was used to observe the cytotoxicity of ST72 against human liver hepatocellular carcinoma (HepG2) cells at 2–1,000 µM concentrations. The graph showed percent cell viability of HepG2, and the experiment was performed in triplicate. Data were shown as mean values ± SD.

Furthermore, the growth inhibitory effect of ST72 as obtained by the *in vitro* study was confirmed *in vivo* using the *P. berghei* ANKA–infected BALB/c mice model, and the antimalarial efficacy of ST72 alone and in combination with a known antimalarial drug chloroquine (CQ) was examined in the rodent malaria model BALB/c infected with *P. berghei* ANKA. Infected mice were administered with ST72 at 2.5 mg/kg and chloroquine at 6 mg/kg doses (Basir et al., 2012; Adebajo et al., 2014; Mazhari et al., 2018): ST72 (2.5 mg/kg), CQ (6 mg/kg), and ST72 + CQ (2.5+6 mg/kg). Thin blood smears were made from *P. berghei*–infected mice to check the parasitemia up to six consecutive days after drug administration (Manohar et al., 2012). Significant reduction in the percentage parasitemia was observed in the ST72-treated group of mice as compared to the untreated control group on day 6 post-treatment. The percentage parasitemia was found to be 13.1% in the ST72 alone group as compared to the untreated control wherein the percent parasitemia was 26.3% on day 6 post-treatment. Co-administration of ST72 with CQ gave 6.4% parasitemia that is less than parasitemia 9.6%, found in the CQ alone group (Figure 5A (i)). The severity of malaria infection may lead to changes in vascular permeability in blood–brain barrier (BBB) in treated mice (Jesús and Spadafora, 2018). Evans blue dye was used to assess any such changes in treated mice as compared to control. Intense coloration of Evans blue dye in control mice as compared to ST72-treated mice showed that the compound did not cause any noticeable changes in BBB and is protective against malarial parasites (Figure 5A (iii)). Survival of group of mice was observed up to 25 days post-infection. Mean survival time (MST) showed improvement in the mortality rate in case of ST72 2.5 mg/kg (MST = 11.5 days), CQ 6 mg/kg (MST = 14.5 days), and combination (MST = 17.5 days) as compared to control (MST = 7.5 days) (Figure 5A (ii)). The graph is plotted as percentage mean survival time. Therefore, we state that the combination of ST72 with CQ resulted in improved growth inhibitory activity than the individual drug. Our *in vivo* data corroborate with the antimalarial potential of ST72 as observed by *in vitro* studies.

#### 3.2.8 Hemolytic and Cytotoxicity Activity

Spectrophotometric analysis was done to see the effect of ST72 on human red blood cells (*h*RBCs) and human liver hepatocellular carcinoma cells (HepG2) (Malik et al., 2019; Saigal et al., 2021). Up to 125 µM concentration, no significant lysis in RBCs was observed by ST72, whereas 0.15% saponin-treated RBCs showed 100% hemolysis (Figure 5B (i)). Compound ST72 did not affect the viability of HepG2 cells because these compounds are non-cytotoxic to human cells in the concentration range tested. The effect of ST72 treatment at 2–1,000 µM concentrations on HepG2 cell viability was determined by the MTT assay (Wani et al., 2019). The result shows the percent survival of HepG2 cells at 500 µM with more than 84% cells being viable (Figure 5B (ii)). The experiment was done in triplicate. Data were expressed as mean values ± SD.

## 4 Discussion

The increasing resistance to current antimalarials demands development of new chemotherapeutic interventions. Understanding the molecular mechanisms that control malarial parasite growth and progression in host erythrocytes might lead to identification of key targets for development of effective antimalarials. In malarial parasites, cysteine proteases like falcipains are essential enzymes involved in hemoglobin degradation and heme detoxification (Rosenthal, 2020). Out of three important falcipains present in the parasite, falcipain-2 is the principal enzyme in trophozoites, majorly responsible for hemoglobin hydrolysis, and therefore a potential drug target (Sijwali et al., 2001b, 3). *P. falciparum* orthologs of falcipains found in rodent malarial parasite *P berghei* such as berghepain-1 and -2 share structural homology with that of falcipains (Ramjee et al., 2006). Small molecules and inhibitors that bind to FP2 and FP3 could efficiently target the cysteine proteases in *P. berghei*. Therefore, in our *in vivo* study, we observed a significant reduction in parasite growth with ST72 which is an inhibitor of FP2 in *P. falciparum* (Hopp et al., 2017). Development of inhibitors targeting *falcipain-2* is an effective therapeutic intervention against malaria. In the present study, we have screened the natural compounds’ library of ZINC database and identified three hit compounds NT23, ST72, and NT18, by structure-based virtual screening methods on the basis of their interaction with FP2 (Neves et al., 2018). We found that these compounds had highest binding affinities with FP2 as compared to FP3 and bound at the critical residues of the FP2 binding pocket. The FP2 binding site located at the main catalytic pocket of FP2 is critical for its functional activity. Therefore, the interaction analysis suggests that NT23, ST72, and NT18 occupy the same position where known inhibitor E64 binds. Together, the results of *in silico* screening studies and analysis supported that computational approaches would play an essential role in the identifications of potential therapeutic molecules to address various complex diseases, including malaria (Fawad Ali et al., 2021). In order to determine the effect of these compounds on FP2 and FP3 enzyme activity, the recombinant FP2 and FP3 were purified from *E. coli* and purified proteins were taken in the enzyme activity assay where the different concentrations of compounds were used. The activity assay includes the known fluorogenic substrate Z-Phe-Arg-AMC which has catalytic sites, cleaved by the enzyme falcipain, and the fluorescence is measured by a fluorospectrometer. Inhibition of fluorescence in the presence and absence of compounds leads to inhibitory activity. The analysis of specificity of falcipain-2 for these compounds revealed that ST72 has highest effect on the FP2 enzyme activity.

Drug discovery approaches against falcipains as antimalarial targets look for stable and strong interaction of these proteins with small molecules of interest (Kerr et al., 2009b). Here, we employed SPR technique that monitors the binding of analytes with macromolecules in real time and gives quantitative response with good sensitivity (Jain et al., 2020a). Highest binding affinity of ST72 was observed for FP2 as compared to FP3 in the SPR study suggesting that ST72 is specific for cysteine protease FP2. Inhibitors of falcipain-2/3 inhibit the growth of malarial parasite as these enzymes are involved in hemoglobin hydrolysis and detoxification of heme (Aratikatla et al., 2021). Quantification of free monomeric heme obtained from hemozoin showing less heme production in the presence of ST72 further indicated reduction in hemoglobin degradation and hence indicated the specificity of compound ST72 for FP2. Different classes of falcipain inhibitors have been examined for their growth inhibitory potency on *Plasmodium* by *in vitro* testing (Li et al., 2009; Marco and Coterón, 2012; Mugumbate et al., 2013; Musyoka et al., 2016). The naturally inspired compounds identified from natural compound databases have drawn much attention due to their curative potential in a number of diseases. These compounds have been shown to be effective as anti-cancer, anti-inflammatory, and anti-viral agents (Mohammad et al., 2019; Abdallah et al., 2021). The *in vitro* growth assay demonstrated potent antimalarial activity of NT23, ST72, and NT18 in chloroquine-sensitive and -resistant strains of *P. falciparum*. It is noteworthy that compound ST72 was found to be most effective in both chloroquine-sensitive and -resistant strains with EC_50_ in lower micromolar ranges (Anju Singh et al., 2020). Previous studies demonstrate that inhibitors of cysteine proteases impede intraerythrocytic development of malarial parasites (Rosenthal et al., 1988). The assessment of sensitivity of different asexual stages to ST72 treatment by the stage-specific assay indicated increased activity of the compound at trophozoite stage. At metabolically active trophozoite stage, parasites digest hemoglobin within digestive vacuoles, which is the process mediated by falcipains. Small molecules or compounds targeting activity of these enzymes would be potential drug candidates. Herein, we showed the stage-specific effect of ST72 on growth and development of parasites at trophozoite stage, indicating this compound targets falcipain enzymes in the parasite. In regard to increasing resistance to available drugs for malaria, there is a need to identify and optimize new drug combinations for treatment of malaria. With the aim of profiling the combinatorial effect of ST72, the growth inhibitory effect of ST72 in association with known antimalarial chloroquine was assessed. The compound ST72, which is identified as a potent FP2 inhibitor in association with chloroquine, exhibited synergistic inhibitory effect on parasite growth. Together, our results facilitate identification of efficacious drug candidates with potent antimalarial effect. The *in vitro* enzyme inhibition assay of ST72 showed lower potency in the biochemical assay as compared to the *in vitro* inhibition assay against the parasite. We have observed significant antimalarial potency of ST72 in growth assays. This could be explained by the stage-dependent expression of the protein in parasites and high concentrations of inhibitors used in the experimental conditions *in vitro*. However, in the case of biochemical enzyme inhibition assay, we use abundant recombinant protein and varying concentrations of inhibitors, which might be responsible for the difference in potency of compounds. We have demonstrated that ST72 specifically binds to and inhibits the activity of FP2; however, off-target effects of the compounds in the cell-based assay cannot be ignored here.

Evaluation of *in vivo* efficacy of compounds as antimalarials is necessary to find new leads for preclinical studies in view of drug development. The assessment of *in vivo* efficacy of ST72 alone or in combination with chloroquine against murine malaria indicates that the compound effectively inhibits parasite growth. Moreover, our results demonstrated that ST72 is not hemolytic and is devoid of any cytotoxicity even at higher concentrations. ST72 showed promising *in vitro* antimalarial activity against both CQ^S^ and CQ^R^ strains and demonstrated potent *in vivo* efficacy as well.

Taken together, our work supports that the FP2 enzyme is required for the hemoglobin hydrolysis in the trophozoite stage and therefore a key target for antimalarial therapy. We identified a novel naturally inspired lead compound ST72 that proved to be a potent inhibitor of FP2, effectively blocking malarial parasite growth and development at sub-micromolar concentrations without any cytotoxic effects. Incontestably, amalgamation of *in silico*, *in vitro*, and *in vivo* approaches speeds up the process of discovery of novel antimalarial compounds.

## 5 Conclusion

Targeting FP2 with natural compounds is an attractive strategy to combat malaria. FP2 is known as a heme-metabolizing enzyme responsible for initiating the degradation of host erythrocyte hemoglobin inside the food vacuole of the parasite. There are limited numbers of drugs available to kill *Plasmodium*, and this warrants the need to develop better antimalarial drugs. We employed a structure-based drug discovery approach and identified three natural compounds NT23, ST72, and NT18, showing appreciable binding affinity with FP2 and drug-like properties followed by *in vitro* and *in vivo* validation for *P. falciparum* inhibitors were pursued. Finally, the screening results obtained were validated experimentally by testing the most promising hits in an *in vitro* biochemical assay against the FPs and cultured *P. falciparum*. Hit compounds were tested against sensitive and resistant strains of *P. falciparum.* The study identified ST72 as the most potent among all against 3D7 and RKL-9 strains with EC_50_ values 2.8 and 6.7 µM, respectively. A defect in parasite growth was observed as the development of parasites was arrested at schizont stage with EC_50_ value 17.7 µM when treated for 6 hrs at trophozoite stage. Cytotoxicity results showed that 84% of HepG2 cells were viable even at 500 µM inhibitor concentration, and the hemolytic assay showed less than 5% of non-toxic nature of the tested inhibitors. The *in vivo* antimalarial efficacy on the murine malaria model of ST72 was evaluated, which displayed significant parasitemia reduction up to 5 days post-treatment. In conclusion, the potential inhibitor ST72 identified from the ZINC database library might be a significant potential lead to develop selective inhibitors of FP2 for the therapeutic management of malaria after required experimentation.

## Data Availability

The contributions presented in the study are included in the research article/Supplementary Material, and further inquiries can be directed to the corresponding authors.
