# Supplementary material for: Target-Based Virtual Screening of Natural Compounds Identifies a Potent Antimalarial With Selective Falcipain-2 Inhibitory Activity
Source: Front Pharmacol. 2022 Apr 6;13:850176. doi: 10.3389/fphar.2022.850176 (PMC9020225; doi:10.3389/fphar.2022.850176)
Supplement: Supplementary file 1 [file DataSheet1.docx]

**Supplementary Information**

**Target-based virtual screening of natural compounds identifies a potent antimalarial with selective falcipain-2 inhibitory activity**

Amad Uddin^1,2^, Sonal Gupta^2^, Taj Mohammad^3^, Diksha Shahi^2^, Afzal Hussain^4^, Mohamed F. Alajmi^4^, Hesham R. El-Seedi^5^, Imtaiyaz Hassan^3^, Shailja Singh^2^*, Mohammad Abid^1^*

*^1^Medicinal Chemistry Laboratory, Department of Biosciences, Jamia Millia Islamia, Jamia Nagar, New Delhi, 110025, India*

*^2^Special Centre for Molecular Medicine, Jawaharlal Nehru University, New Delhi, 110067, India*

*^3^Center for Interdisciplinary Research in Basic Sciences, Jamia Millia Islamia, Jamia Nagar, New Delhi-110025, India*

*^4^Department of Pharmacognosy, College of Pharmacy, King Saud University, Riyadh 11451, Saudi Arabia*

*^5^Department of Medicinal Chemistry, Uppsala University, Biomedical Centre, Box 574, 75123, Uppsala, Sweden*

^✉^To whom correspondence should be addressed**:** [mabid@jmi.ac.in](mailto:mabid@jmi.ac.in) (M. Abid), [shailja.jnu@gmail.com](mailto:shailja.jnu@gmail.com) (S. Singh).

**Table of Contents**

1. Docking score of top 30 screened compounds and FP2 and FP3 TS1-S2
2. Docking score of top 10 compounds against FP2. TS3
3. ADMET properties of those compounds which selected finally based on interaction and selectivity. TS4
4. Selected compounds interface the complexes individually FS1-FS2
5. Surface Plasmon Resonance of NT23 and NT18 against FPs. FS3

| **S. No.** | **Compound ID** | **Affinity (kcal/mol) with FP2** | **pKi** | **Torsional Energy** | **Ligand Efficiency (kcal/mol/non-H atom)** |
| --- | --- | --- | --- | --- | --- |
| 1. | ZINC03845566 | -11.5 | 8.43 | 0 | 0.3026 |
| **2.** | **ZINC12900664 (ST72)** | **-10.1** | **7.41** | **1.5565** | **0.2658** |
| 3. | ZINC05434077 | -10.1 | 7.41 | 1.5565 | 0.273 |
| 4. | ZINC12898980 | -10.1 | 7.41 | 1.5565 | 0.273 |
| 5. | ZINC08877717 | -10 | 7.33 | 0.6226 | 0.2703 |
| 6. | ZINC05433942 | -10 | 7.33 | 1.5565 | 0.2632 |
| **7.** | **ZINC05434062 (NT23)** | **-10** | **7.33** | **1.8678** | **0.2564** |
| 8. | ZINC08877879 | -9.9 | 7.26 | 0.6226 | 0.275 |
| 9. | ZINC08918500 | -9.8 | 7.19 | 1.2452 | 0.2579 |
| 10 | **ZINC05434059 (NT18)** | **-9.8** | **7.04** | **1.8678** | **0.2595** |
| 12. | ZINC12296715 | -9.8 | 7.19 | 1.5565 | 0.2513 |
| 12. | ZINC12604545 | -9.8 | 7.19 | 1.5565 | 0.2649 |
| 13. | ZINC08299978 | -9.7 | 7.11 | 0.6226 | 0.2694 |
| 14. | ZINC12890378 | -9.7 | 7.11 | 0.6226 | 0.2694 |
| 15. | ZINC08300078 | -9.7 | 7.11 | 0.9339 | 0.2771 |
| 16. | ZINC05434050 | -9.7 | 7.11 | 1.8678 | 0.2622 |
| 17. | ZINC04222182 | -9.6 | 7.04 | 0.9339 | 0.2595 |
| 18. | ZINC04236421 | -9.6 | 7.04 | 0.9339 | 0.2526 |
| 19. | ZINC08299197 | -9.6 | 7.04 | 1.8678 | 0.24 |
| 20. | ZINC08765217 | -9.5 | 6.97 | 0.6226 | 0.3065 |
| 21. | ZINC02125216 | -9.5 | 6.97 | 0.9339 | 0.2794 |
| 22. | ZINC08300223 | -9.5 | 6.97 | 0.9339 | 0.2568 |
| 23. | ZINC02120544 | -9.4 | 6.89 | 0.9339 | 0.2938 |
| 24. | ZINC08300065 | -9.4 | 6.89 | 0.9339 | 0.2611 |
| 25. | ZINC08877295 | -9.4 | 6.89 | 0.9339 | 0.2611 |
| 26. | ZINC12604806 | -9.4 | 6.89 | 0.9339 | 0.2611 |
| 27. | ZINC12890485 | -9.4 | 6.89 | 0.9339 | 0.2541 |
| 28. | ZINC08792598 | -9.4 | 6.89 | 1.5565 | 0.2611 |
| 29. | ZINC08792130 | -9.3 | 6.82 | 1.5565 | 0.2447 |
| 30. | ZINC12865513 | -9.1 | 6.67 | 1.2452 | 0.2395 |

**Table S1** Docking score screened compounds done by InstaDock towards FP-2 along with compounds code. List of selected compounds based on their binding affinity towards FP2.

**Table S2** Docking score screened compounds done by InstaDock towards FP-3 along with compounds code. List of selected compounds based on their binding affinity towards FP3.

| **S. No.** | **Compound ID** | **Affinity (kcal/mol) with FP3** | **pKi** | **Torsional Energy** | **Ligand Efficiency (kcal/mol/non-H atom)** |
| --- | --- | --- | --- | --- | --- |
| 1. | ZINC03845566 | -9.4 | 8.8 | 0 | 0.2958 |
| 2. | ZINC12890378 | -9.2 | 7.41 | 0.6226 | 0.2806 |
| 3. | ZINC12890485 | -9.2 | 7.41 | 0.9339 | 0.273 |
| 4. | ZINC05433942 | -9.1 | 7.41 | 1.5565 | 0.2658 |
| 5. | ZINC08877717 | -9 | 7.33 | 0.6226 | 0.2703 |
| 6. | ZINC04236421 | -9 | 7.33 | 0.9339 | 0.2632 |
| 7. | ZINC08792130 | -9 | 7.33 | 1.5565 | 0.2632 |
| 8. | ZINC12898980 | -9 | 7.33 | 1.5565 | 0.2703 |
| **9.** | **ZINC12900664 (ST72)** | **-8.9** | **7.26** | **1.8678** | **0.2538** |
| 10. | ZINC05434077 | -8.8 | 7.19 | 1.5565 | 0.2649 |
| 11. | ZINC08300223 | -8.7 | 7.11 | 0.9339 | 0.2622 |
| **12.** | **ZINC05434062 (NT23)** | **-8.7** | **7.11** | **1.5565** | **0.2553** |
| 13. | ZINC08299197 | -8.7 | 7.11 | 1.8678 | 0.2425 |
| 14. | ZINC08877879 | -8.6 | 7.04 | 0.6226 | 0.2667 |
| 15. | ZINC08300065 | -8.6 | 7.04 | 0.9339 | 0.2667 |
| 16. | ZINC05434050 | -8.6 | 7.04 | 1.8678 | 0.2595 |
| 17. | ZINC08765217 | -8.5 | 6.97 | 0.6226 | 0.3065 |
| 18. | ZINC12604806 | -8.5 | 6.97 | 0.9339 | 0.2639 |
| 19. | ZINC12296715 | -8.5 | 6.97 | 1.5565 | 0.2436 |
| 20. | ZINC02125216 | -8.4 | 6.89 | 0.9339 | 0.2765 |
| 21. | ZINC04222182 | -8.4 | 6.89 | 0.9339 | 0.2541 |
| 22. | ZINC08877295 | -8.3 | 6.82 | 0.9339 | 0.2583 |
| 23. | ZINC12604545 | -8.3 | 6.82 | 1.5565 | 0.2514 |
| 24. | ZINC08792598 | -8.2 | 6.75 | 1.5565 | 0.2556 |
| 25. | ZINC02120544 | -8.1 | 6.67 | 0.9339 | 0.2844 |
| 26. | ZINC08300078 | -8.1 | 6.67 | 0.9339 | 0.26 |
| **27.** | **ZINC05434059 (NT18)** | **-8.1** | **6.67** | **1.8678** | **0.2459** |
| 28. | ZINC08918500 | -7.7 | 6.38 | 1.2452 | 0.2289 |
| 29. | ZINC12865513 | -7.7 | 6.38 | 1.2452 | 0.2289 |
| 30. | ZINC08299978 | -7.4 | 6.16 | 0.6226 | 0.2333 |

**Table S3** List of selected 10 compounds based on their binding affinity towards FP2.

| **S. No.** | **Compound ID** | **Structure of Compounds** | **Affinity (kcal/mol)** | **Ligand Efficiency (kcal/mol/non-H atom)** |
| --- | --- | --- | --- | --- |
| 1. | ZINC03845566 | 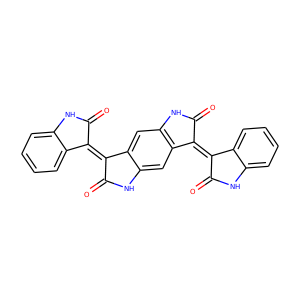 | -11.5 | 0.30 |
| 2. | ZINC12900664 **(ST72)** | 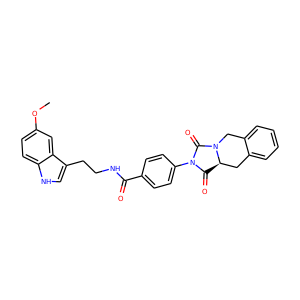 | -10.1 | 0.27 |
| 3. | ZINC05434077 | 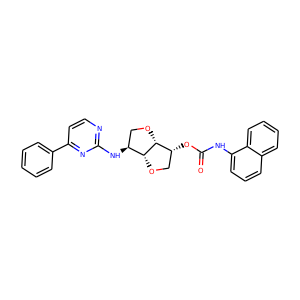 | -10.1 | 0.27 |
| 4 | ZINC12898980 | 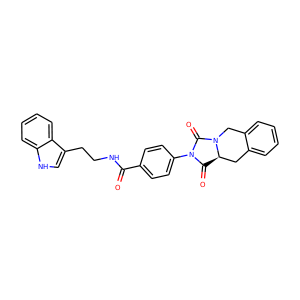 | -10.1 | 0.27 |
| 5. | ZINC08877717 | 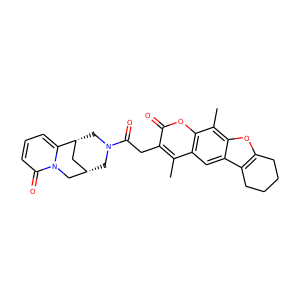 | -10.0 | 0.27 |
| 6. | ZINC05433942 | 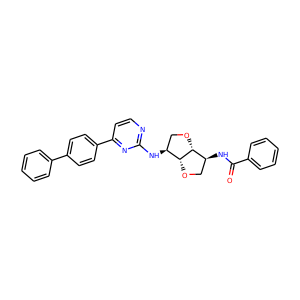 | -10.0 | 0.26 |
| 7. | ZINC5434062 **(NT23)** | 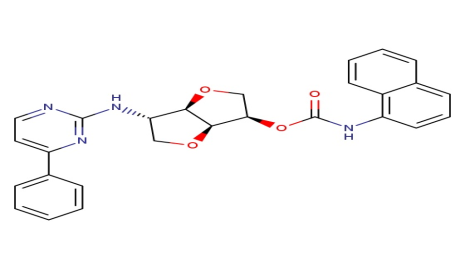 | -10.0 | 0.26 |
| 8. | ZINC08877879 | 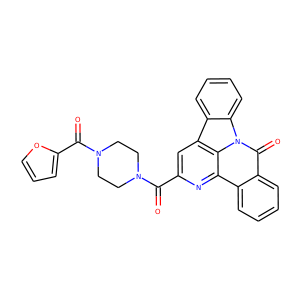 | -9.9 | 0.28 |
| 9 | ZINC08918500 | 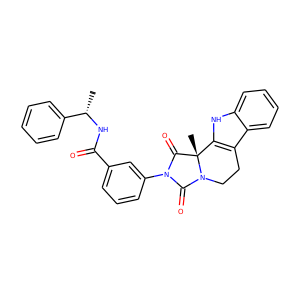 | -9.8 | 0.31 |
| 10. | ZINC05434059 **(NT23)** | 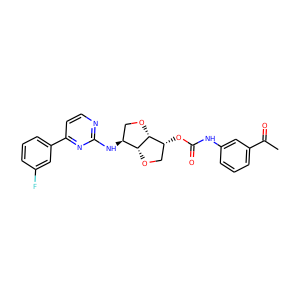 | -9.8 | 0.26 |
| 11. | E64 |  | -5.3 | - |

**Table S4** Absorption, Distribution, Metabolism, Excretion, and Toxicity (ADMET) properties of the selected compounds along with CCL OWB (E64).

| Compound ID | Absorption | | Distribution | Metabolism | Excretion | Toxicity |
| --- | --- | --- | --- | --- | --- | --- |
|  | ***GI***  ***Absorption***  ***(%)*** | ***Water Solubility***  ***(log mol/L)*** | ***BBB/CNS permeation*** | ***CYP2D6***  ***Inhibitor*** | ***OCT2 substrate*** | ***Skin sens.*** |
| NT23 | 82.44 | -3.65 | No | No | No | No |
| ST72 | 99.05 | -2.70 | No | No | Yes | No |
| NT18 | 80.13 | -3.25 | No | No | Yes | No |
| E64 | 94.66 | -3.45 | No | No | Yes | No |

**Figure S1 (A)** *Cartoon representation showing* the interfaces of the predicted complexes individually with *the docked E64* ***(Ai)*** *and NT-23* ***(Bi)*** *interacting to the binding site residues of FP2. Surface potential view of FP2 binding pocket occupied by E64* ***(Aii)*** *and NT-18* ***(Bii)***

**Figure S2 (A)** *Cartoon representation showing* the *docked ST-72* ***(Ai)*** *and NT-18* ***(Bi)*** *interacting to the binding site residues of FP2. Surface potential view of FP2 binding pocket occupied by ST-72* ***(Aii)*** *and NT-18* ***(Bii)***

**Figure S3**: Sensograms representing interaction of FP2 and FP3 with NT23 and NT18 at different concentrations (12.5-400µM) by surface plasmon resonance.

**Growth inhibition assay of compounds 5A – 5L**
